# Case report: What course to follow when left bundle branch pacing encounters acute myocardial infarction?

**DOI:** 10.3389/fcvm.2022.969192

**Published:** 2022-10-03

**Authors:** Xiaojiang Zhang, Yanzhuo Ma, Leisheng Ru, Dongmei Wang, Jie Li, Shuying Qi

**Affiliations:** 980th Hospital of the Joint Logistic Support Force of PLA, Shijiazhuang, China

**Keywords:** left bundle branch pacing (LBBP), acute myocardial infarction (AMI), AV block, pacemaker dysfunction, the pacing threshold

## Abstract

Compared with traditional right ventricular apical pacing, His-bundle pacing (HBP) provides more physiologic pacing by activating the normal conduction system. However, HBP has some limitations including higher pacing thresholds. In addition, disease in the distal His-Purkinje system may prevent the correction of abnormal conduction. Left bundle branch pacing (LBBP) may overcome these disadvantages by providing lower pacing thresholds and relatively narrow QRS duration that improve cardiac function. Here, we describe a rare case of a transient loss of ventricular capture due to acute anterior wall myocardial infarction in an LBB-paced patient. With the improvement of the ischemia, the function of the pacemaker partly recovered. We review the adaptations, advantages, and limitations, and long-term safety of LBBP.

## Case presentation

### Treatment of AMI

**Figure 1:**
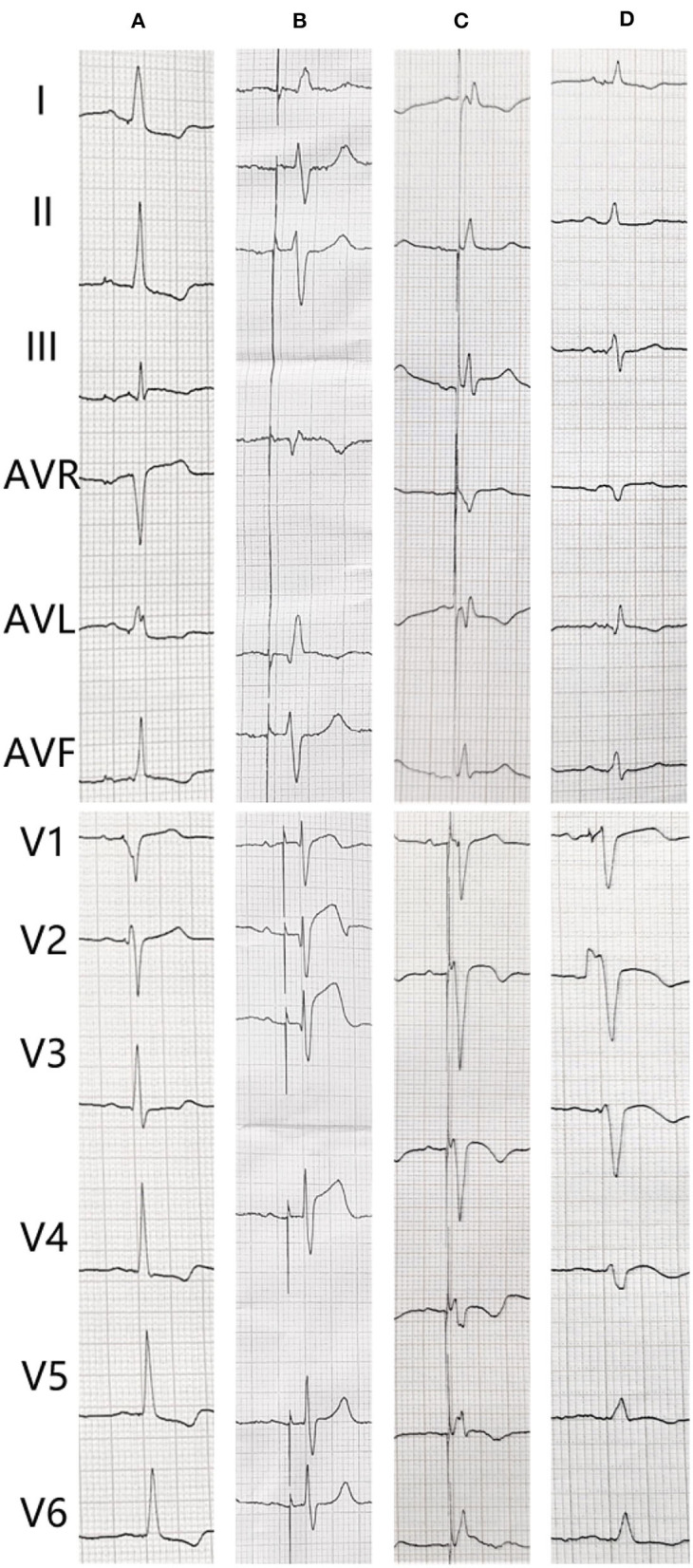
The electrocardiogram (ECG) changes of the patient. **(A)** After pacemaker implantation. **(B)** The transient loss of capture when acute myocardial infarction. **(C)** Second day after coronary angiography. **(D)** After percutaneous coronary intervention.

**Figure 2:**
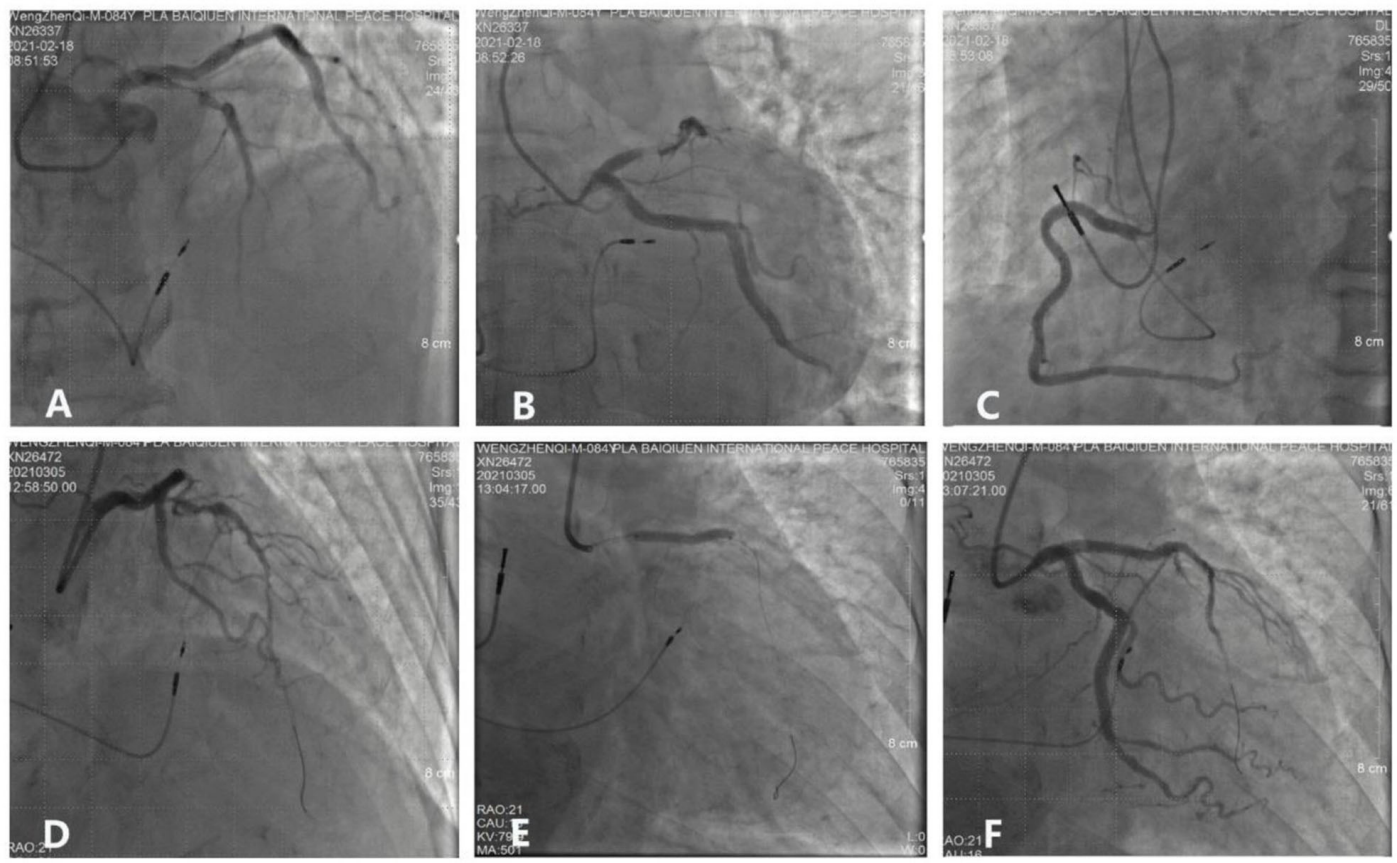
Process and visualization of percutaneous coronary intervention (PCI). **(A,B)** A fixed stenosis in the left anterior descending artery. **(C)** No severe stenosis in the right coronary. **(D)** NS guidewire was placed in the targeted artery. **(E)** A stent was placed in the proximal segment of the left anterior descending artery. **(F)** No residual stenosis after stent implantation.

An 82-year-old man who was treated with LBBP in 2018 for atrioventricular (AV) conduction disorder was admitted to the Chest Pain Center of the 980th Hospital of the Joint Logistic Support Force of the People's Liberation Army (PLA) in 2021. Based on typical acute chest pain, the elevation of the ST segment in leads V1–V4 of the electrocardiogram (ECG) (Figure 1B), and positive cardiac troponin I (12.34 ng/ml), the patient was diagnosed with an acute anterior wall myocardial infarction (AMI). According to the AMI treatment guidelines, the patient underwent emergency coronary angiography. In his left anterior descending (LAD) artery, there was thrombosis with fixed stenosis in the proximal segment, whereas no significant stenosis was observed in the left circumflex artery and the right coronary artery (Figures 2A,B). Because thrombolysis resulted in grade 3 flow in the distal part of the LAD, we prepared to perform percutaneous coronary intervention (PCI) after initiating standard drug therapy that included dual antiplatelets, statin, metoprolol, and diuretics. A subsequent laboratory test revealed no pathologic features except for 88.3% of white blood cells being neutrophils (40–75%), creatine kinase (CK) of 2,179 U/l (50–301 U/l), CK isoenzyme of 140 U/l (0–24 U/l), creatinine of 117 μmol/l (57–111 μmol/l), and d-dimer of 1.227 mg/l (0–0.243 mg/l), which led to a diagnosis of AMI. A computed tomography (CT) scan of the lung revealed bilateral pneumonia and hydrothorax that was related to the heart failure. Echocardiography revealed a left ventricular end-diastolic diameter of 56 mm, ejection fraction of 42%, interventricular septal thickness of 11.8 mm, and decreased diastolic function. The second day after AMI, the ST segments on his ECG declined toward the baseline value with inverted T waves in the anterior leads (Figure 1C).

### Adjustment of LBBP parameters

**Figure 3:**
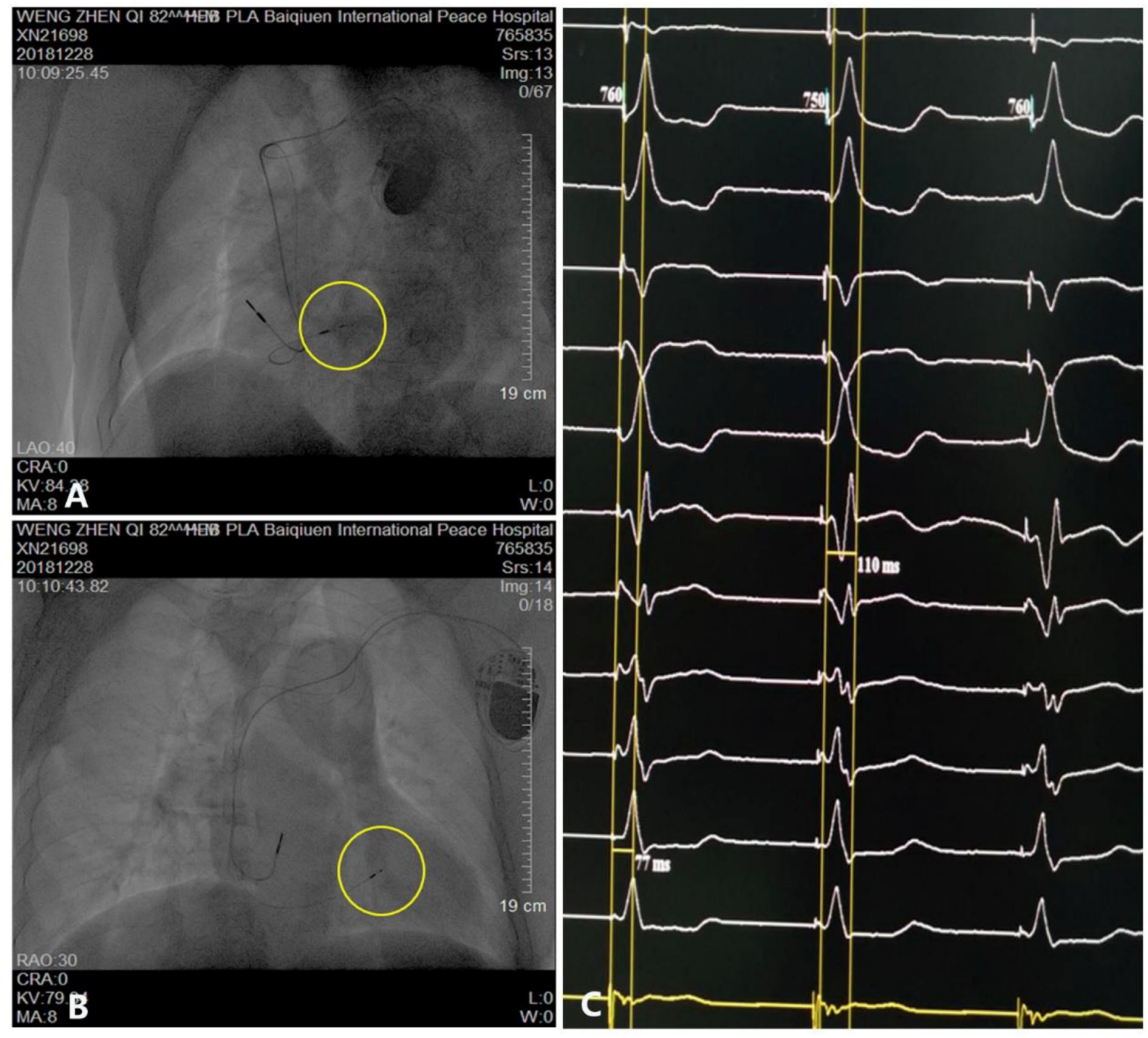
Parameters and visualization of pacemaker implantation. **(A,B)** Pacing electrode of the left bundle branch (yellow circle). **(C)** Electrocardiogram showing a QRS duration of 110 ms.

The patient underwent LBB pacemaker implantation for second-degree type II AV block according to the treatment guidelines. A 3,830 lead (Medtronic, Inc. Minneapolis, MN, US) was advanced and positioned *via* a transventricular septal approach (Figures 3A,B). The procedure was performed successfully, with a pacing threshold of 0.5 V/0.4 ms and a QRS duration of 110 ms on the ECG (Figures 1A,C, 3C). The pacemaker had a normal function in subsequent examinations until the occurrence of AMI (Table 1). Compared with the previous ECG, there was a transient loss of capture and an increase in the LBBP threshold at the time of AMI (Figure 1B; Table 1). Pectoral muscle twitching was noted which indicated some changes in the electrical performance of the pacing system. The pacemaker and electrode were immediately tested; the ventricular pacing threshold had increased to 5 V/0.4 ms (Table 1) whereas that of the right atrium remained stable compared with the value recorded at implantation. After parameters adjustment, the symptoms disappeared and the threshold decreased to 3.5 V/0.4 ms. Three days after AMI, in the absence of additional acute ischemia, the pacing threshold had decreased to 2.5 V/0.4 ms and was stable. The LAD PCI was performed after 2 weeks (Table 1). A 3.5 × 28 mm drug-coated stent (Boston Scientific, Marlborough, Massachusetts, US) was placed in the proximal segment of the LAD (Figures 2D–F). A routine test showed that the pacing threshold had decreased to 2 V/0.4 ms (Figure 1D, Table 1).

**Table 1:** Pacemaker pacing and sensing thresholds of the right atrium and left bundle branch at the time of implantation and acute myocardial infarction and during follow-up.

	**2018.12.18**	**2021.02.19**	**2021.02.21**	**2021.03.05**	**2021.03.12**	**2021.04.27**	**2022.05.11**
	**Pacemaker implantation**	**AMI**	**3 days after AMI**	**2 weeks after AMI**	**3 weeks after AMI**	**2 months after AMI**	**1.4 years after AMI**
LBB pacing threshold	0.5 V/0.4 ms	5.0 V/0.4 ms	2.5 V/0.4 ms	2.0 V/0.4 ms	2.0 V/0.4 ms	2.0 V/0.4 ms	1.75 V/0.4 ms
LBB sensing threshold	10 mV	11–15.6 mV	15.6 mV	Dependence	Dependence	Dependence	Dependence
RA pacing threshold	0.5 V/0.4 ms	0.25 V/0.4 ms	0.25 V/0.4 ms	0.25 V/0.4 ms	0.25 V/0.4 ms	0.25 V/0.4 ms	0.25 V/0.4 ms
RA sensing threshold	5.0 mV	2.8 mV	2.8 mV	4.0 mV	4.0–5.6 mV	2.8 mV	4.0–5.6 mV

AMI, acute myocardial infarction; LBB, left bundle branch; RA, right atrium.

### Outcome and follow-up

The patient recovered well and was discharged. After more than 1.4 years of follow-up, the patient showed good recovery with no complications and a septal thickness of 9 mm. The ventricular pacing threshold remained relatively steady with a slight decrease to 1.75 V/0.4 ms (Table 1).

## Discussion

To overcome the limitations of right ventricular apical pacing (RVAP) such as electrical and mechanical dysynchrony and a high risk of heart failure (1), two physiologic stimulation techniques have been employed—namely, HBP and LBBP (2). Although HBP has some demonstrated benefits (3) such as specific activation of the conduction system and has been broadly adopted, it has certain drawbacks including the difficulty of identifying the precise location of the His bundle, unstable pacing threshold, or lead dislodgement rate of 5–10%, large atrial signals, or low R-wave amplitude that complicate pacing management, and heart block distal to the pacing cite (4). The LBBP is a novel technique for stimulating the cardiac conduction system. Direct LBBP, another physiologic pacing method, was first used during pacemaker implantation to restore the impaired His-Purkinje conduction system in a patient with heart failure and LBB block (5). From 2018 to the present, our center has performed more than 100 LBBP procedures and has described the advantages of LBBP such as a lower and more stable pacing threshold, reduced heart failure rate, narrow QRS duration, high success rate, and few complications. The LBBP is gaining rapid acceptance among clinicians (6).

In this report, we described a patient with AV block and a high percentage of right ventricular pacing rate that was successfully treated with LBBP. After the implantation in 2018, the threshold of the pacemaker remained stable and was similar to that observed at the time of implantation, and the patient was asymptomatic without heart failure for more than 2 years till the year 2021.

Many articles and systematic reviews have demonstrated the effectiveness of LBBP (3), which was confirmed in our patient. However, some researchers have cautioned that additional studies are needed to validate the safety of LBBP (6) as some complications have been reported including septal perforation and thromboembolism (7), septal arterial injury (8), and lead dislodgement (9). Our patient was diagnosed with an acute anterior wall myocardial infarction 2 years after LBBP, and emergency coronary angiography revealed LAD stenosis in the proximal segment with transient loss of LBBP capture in the ECG. During testing the LBB pacing threshold of had increased to 5 V/0.4 ms, which was related to the myocardial ischemia in the septum. To investigate whether the infarction scar also contributed to this increase, we further tested the pacemaker 3 days after the AMI and found that the threshold had decreased to 2.5 V/0.4 ms. After standard drug therapy for 2 weeks and selective PCI for the LAD stenosis, the pacing threshold further declined to 2 V/0.4 ms, likely due to the additional improvement of the myocardial blood supply. This suggests that ischemia played a pivotal role in the threshold change. As for the cardiac scar, there was no evidence that it was a contributing factor. After discharge, the patient was followed up for more than 1.4 years, during which time the pacing threshold remained almost the same as that recorded at the time of PCI, with only a minor decline after 1.4 years.

Compared with RVAP and HBP, LBBP has technical advantages, especially in terms of physiologic pacing, but there are still some aspects that warrant consideration (10). First, it is essential to strictly comply with the LBBP operation procedure, which is summarized as follows: (1) determine the initial LBBP site; (2) introduce a pacing lead into the interventricular septum (IVS) and reach the LBB area; (3) assess the lead depth and confirm LBB capture; (4) remove the sheath and provide slack; and (5) program the pulse generator (10). Second, after assessing cardiac ischemia and scarring of the myocardium (especially the septum), it is important to avoid pacing dysfunctions such as a high threshold or unstable electrode. Coronary computed tomography angiography and magnetic resonance imaging of the heart can provide valuable information. Third, the patient should be closely followed up to monitor pacemaker function so that pacing problems can be detected and corrected in a timely manner.

Short-term clinical outcomes of physiologic pacing, especially LBBP, have been promising (11, 12). Nonetheless, prospective clinical trials and mechanistic studies are needed to better understand this technique and improve its safety and reliability.

## Conclusion

The IVS ischemia may occur more frequently than with RVAP in patients with LBBP, making them more prone to loss of capture, which should be taken into account when selecting the mode of pacing.

## Data availability statement

The original contributions presented in the study are included in the article/supplementary material, further inquiries can be directed to the corresponding author/s.

## Ethics statement

The studies involving human participants were reviewed and approved by the Ethics Committee of 980th Hospital of the Joint Logistic Support Force of PLA, Hebei Medical University. Written informed consent to participate in this study was provided by the In review participants' legal guardian/next of kin. Written informed consent was obtained from the minor(s)' legal guardian/next of kin for the publication of any potentially identifiable images or data included in this article.

## Author contributions

XZ, SQ, and DW were responsible for study design and manuscript preparation. XZ, YM, and JL performed clinical data acquisition. LR contributed to the operation of PCI. XZ wrote the manuscript. All the authors contributed to the article and approved the submitted version.

## Funding

This work was supported by the Key Project of Medical Science Research in the Hebei Province (grant no. 20200232).

## Conflict of interest

The authors declare that the research was conducted in the absence of any commercial or financial relationships that could be construed as a potential conflict of interest.

## Publisher's note

All claims expressed in this article are solely those of the authors and do not necessarily represent those of their affiliated organizations, or those of the publisher, the editors and the reviewers. Any product that may be evaluated in this article, or claim that may be made by its manufacturer, is not guaranteed or endorsed by the publisher.
